# The association between daily movement behavior and adjusted handgrip strength in post-menopausal women

**DOI:** 10.3389/fnut.2025.1538002

**Published:** 2025-03-31

**Authors:** Aishah Alzuwaydi, Ghedeir M. Alshammari, Mohammed A. Mohammed, Rizwan Qaisar, M. Azhar Hussain, Shaea A. Alkahtani

**Affiliations:** ^1^Department of Nutrition, College of Agriculture and Nutrition, King Saud University, Riyadh, Saudi Arabia; ^2^Department of Basic Medical Sciences, College of Medicine, University of Sharjah, Sharjah, United Arab Emirates; ^3^Department of Finance and Economics, College of Business Administration, University of Sharjah, Sharjah, United Arab Emirates; ^4^Department of Social Sciences and Business, Roskilde University, Roskilde, Denmark; ^5^Department of Exercise Physiology, College of Sport Sciences and Physical Activity, King Saud University, Riyadh, Saudi Arabia

**Keywords:** sarcopenia, handgrip strength, physical activity, sedentary, post-menopausal women

## Abstract

**Background:**

The factors driving the sarcopenia phenotype in post-menopausal women remain partly elusive. We thus investigated the associations of physical activity, sedentary behavior, and metabolic biomarkers with handgrip strength (HGS) as a marker of probable sarcopenia in Saudi post-menopausal women.

**Methods:**

We recruited 268 post-menopausal women aged 50 years or above. Physical activity was assessed using Global Physical Activity Questionnaire (GPAQ), and body composition was measured with a BIA device. Blood samples were used to measure cholesterol and triglyceride levels. Blood pressure and waist circumference (WC) were measured. HGS was measured using a digital HGS dynamometer and an HGS < 16 kg was used to define probable sarcopenia. We applied the ordinary least squares (OLS) regression approach for the dependent variables HGS, HGS relative to skeletal muscle mass (SMM) and HGS relative to fat-to-mass ratio (FMR).

**Results:**

Physical activity was positively associated with HGS, and sedentary behavior was negatively associated with HGS (*p* < 0.05). HGS relative to body mass index (BMI) and SMM revealed significant negative relationships with WC (*p* < 0.05). Taking into account age and FMR, the association with HGS or HGS/SMM existed for physical activity (*p* < 0.05), sedentary behavior (*p* < 0.01), and WC (*p* < 0.001).

**Conclusion:**

Altogether, we report that high physical activity, low sedentary behavior and low WC are negatively associated with a risk of low HGS among Saudi post-menopausal women.

## Introduction

Aging is associated with chronic health conditions, including age-associated muscle decline, termed sarcopenia, along with hypertension, hyperlipidemia, diabetes, mental health illnesses, dementia, and malnutrition (1, 2). The health issues of aging can be reduced by healthy aging, defined as developing and maintaining the functional ability that enables well-being in old age (3). In 2010, the European Working Group on Sarcopenia in Older People (EWGSOP) defined sarcopenia as a loss in muscle mass and a reduction in muscle function (4, 5). In early 2018, the definition was updated according to scientific and clinical findings over the previous decade. Sarcopenia was diagnosed according to three parameters, including muscle strength, muscle quantity/quality, and physical performance (6).

HGS is a key indicator of an individual’s health condition (7). HGS test is used to assess overall muscle strength with relevance to generalized health (8–10). A low HGS (<16 kg) signifies probable sarcopenia among women and may herald several age-related diseases (11). This cut-off can slightly vary between different countries, such as <17.5 kg in Argentinian women above 50 years (12) and < 18 kg based on the Asia Working Group for Sarcopenia (13). In brief, the European Working Group on Sarcopenia in Old People and The Foundation of the National Institute of Health use the HGS threshold of <16 kg for women for defining probable sarcopenia, and as such it has been widely used by most of the international studies (14).

Sarcopenia and metabolic syndrome are primarily characterized by skeletal muscle wasting and decreased physical performance, generally leading to weakness (15). Given the benefits of exercise and a balanced diet, it is important to encourage the female population to counter the adverse effects of sarcopenia to improve their quality of life. Evidence indicates that body composition, diet, and hormonal changes after menopause may drive the sarcopenia process, and a shift into a positive lifestyle may be beneficial in promoting muscle strength (16). The decrease in the estrogen hormone due to menopause in women contributes to low bone mass density, low muscle mass, low muscle strength, and redistribution of fat in the visceral region (17).

The association between physical activity and sarcopenia is inconclusive. For example, higher frequency of exercise was inversely associated with appendicular lean mass, but the association becomes positive when adjusting for body weight, and the association between physical activity and handgrip strength (HGS) was found only among middle-aged males (18). In a recent up-to-date comprehensive evident review, Sánchez-Sánchez et al. (19) concluded that moderate to vigorous physical activity (MVPA) is associated with a reduced risk of sarcopenia. However, Beaudart (20) commented on this review and showed that the strength of evidence is low and based on cross-sectional design with inconsistencies between studies and low precision of estimates. Furthermore, a recent systematic review stated that the association between sedentary behavior and sarcopenia independently of physical activity is controversial (21). The recent review by Sánchez-Sánchez et al. (19) also reported that there was no evidence of the association between sedentary behavior and risk of sarcopenia. It should be noted that even if sedentary does not have a strong evident relationship with sarcopenia, sedentary behavior is associated with several adverse health aspects that relate to sarcopenia and weakness including risk of falls (22).

Most of the research on sedentary behavior and muscle strength in aging populations comes from Europe and America. In contrast, relevant data from Saudi Arabia is scarce, despite evidence of high sedentary behavior and obesity in the country (23). In addition, Saudi Arabia has a 19% prevalence of sarcopenia (24), significantly higher than the global average of 10–16% (25). These factors may explain the lower HGS observed in Saudi older adults compared to Western populations (26). Notably, menopause is associated with accelerated muscle loss and increased sedentary lifestyle (27, 28). In addition, menopausal transition is accompanied by increased obesity (29), which is an independent risk factor for sarcopenia and muscle weakness (30). Waist circumference (WC), body mass index (BMI), and body fat content are routinely used to assess obesity and fat deposition in the body. Lastly, the loss of muscle strength in old age may precede muscle atrophy, necessitating the need for using adjusted rather than raw HGS in assessing muscle quality (31). HGS is usually adjusted to BMI or skeletal muscle mass (SMM) to evaluate the intrinsic force-generating capacity and quality of skeletal muscle. However, a relevant study in Saudi Arabia remains elusive. Therefore, a regional study in Saudi Arabia is essential to investigate the associations between low HGS, sedentary behavior, and body composition, but such research in Saudi post-menopausal women remains elusive.

We conducted this study to investigate the associations of a sedentary lifestyle and body composition with low HGS in Saudi post-menopausal women. We hypothesized that in Saudi post-menopausal women MVPA is inversely associated with low HGS, the combined ratio between muscle and fat plays a significant role in this association, sedentary behavior could be associated with low HGS.

## Methods

### Participants

This study was conducted at the King Salman Social Center in Riyadh City, Kingdom of Saudi Arabia (KSA). The initial sample was 277 women who started participating in the study; however, nine women were excluded from the sample because they did not complete the blood analysis, so the final sample size was 268 post-menopausal women. Participants were asked to participate in the study through announcements posted on social media, posters, and banners. The main inclusion criteria were to be in the post-menopausal stage with age ≥ 50 years old to avoid the stage of menopause transition, which is defined as the final cessation of menstruation due to the loss of follicular activity of the ovaries, followed by a period of 12 months without any bleeding. Exclusion criteria included women who had received treatment for some diseases such as cancer, renal failure, and celiac, neurological, and liver diseases. All procedures included in this study were approved by the Research Ethics Committee at King Saud University (IRB No. E-19-4470).

### Study design and data collection

The present study is a cross-sectional design which uses a convenient sampling recruitment in a single governmental social center. The study procedure requires from each participant to get only one visit to encourage participation in the study, such that intra-day variability was not applied, and single day measurement is acknowledged which increase the bias of outcomes. Eligible participants were given the procedure of the study and were scheduled to attend the center only once. Data collection included anthropometry, body composition, questionnaires on physical activity, blood markers, and the measure of HGS.

### Anthropometric measure

Height was measured using a stadiometer, and weight was measured using a weight scale. For height measurement, we asked subjects to take off their shoes, socks, and head covers and stand upright on the equipment. BMI is a measurement derived from an individual’s height and weight used as an indicator of obesity [BMI = weight (kg)/height (m^2^)].

WC was measured by requiring subjects to stand upright, breathe naturally, and relax as a measuring tape was wrapped around their abdomen at the level of the navel (32). The tape was neither loose nor tight around the waist, and the value was recorded in centimeters.

### Bioelectrical impedance analysis (BIA)

The body composition analyzer Tanita (MC-780MA) was used. Subjects were previously asked not to have any implants triggering electric current flow, such as a pacemaker. Participants were asked to refrain from eating and drinking at least 4 h prior the measurement, and to not engage in any high intensity exercise in the day before the test, and to have their regular dietary intake in the last 24 h prior the visit. These instructions are implemented in the whole study procedure. After inserting subjects’ information into the device and hearing the ringing sound of the device, they were asked to step on the device barefoot in an upright position. On the second ring, they were asked to grasp the handles with both hands. The whole procedure took less than 20 s (33). Data on body fat and muscle mass were recorded by the researcher into an Excel sheet for statistical analysis.

### Evaluation of physical activity

Physical activity was assessed using the Global Physical Activity Questionnaire (GPAQ), which is approved for use by the World Health Organization (WHO), and provided to our participants in the Arabic version (34). This questionnaire consists of 16 questions and gathers data on three domains: work, commuting/transportation, and free time. It enables examination of the pattern and intensity of daily physical activity and obtains information regarding the amount of daily time spent sitting, and has been validated in Saudi Arabia (35). A certified exercise physiologist conducted the interview face to face with the participants, and filled the questionnaire.

### Metabolic markers

Venous blood (10 mL) was obtained from our study subjects at the center, and were analyzed at the Central Tower Laboratory in Saudi Arabia. The blood samples were analyzed to determine triglycerides (TG) and high-density lipoprotein (HDL). Additionally, systolic and diastolic blood pressure among participants was measured. We did not use a cut-off in the treatment of our data.

### Handgrip strength (HGS)

According to the EWGSOP2, low muscle strength cut-off is defined as <16 kg when using the HGS test in women (6). A HGS dynamometer was used in the current study, which is a simple, fast, reliable, and inexpensive technology (36). Each subject was asked to stand up with their arms in a comfortable position. With the dominant hand pressing the device arm to the top direction, the subject was instructed to exert as much strength as she could. No other body movement was allowed during the procedure.

### Statistical analysis

Given the distributive characteristics of the dependent variables HGS and HGS/SMM, we applied the ordinary least squares (OLS) regression approach (37):


(1) HGS=α+βX+γAge+ε

(2) HGS=α+βX+γFMR+ε

(3) HGS/SMM=α+βX+γAge+ε

(4) HGS/SMM=α+βX+γFMR+ε

e.g., for physical activity and biomarker measurement *X*, we model, respectively, the HGS as well as the standardized HGS (HGS/SMM) controlling, respectively, for age and FMR. *X* thus sequentially includes physical activity, sedentary, triglyceride, HDL, systolic, diastolic, and WC. For instance, in equation (1) when *X* = physical activity, we estimate the relationship between HGS and physical activity while controlling for age. In equation (2) we carry out the same regression, but now controlling for FMR (instead of age). Equations (3, 4) have similar interpretations. *α*, *β*, *γ*, and *ε* is, respectively, equation specific constant, effect of *X*, effect of the control age or FMR, and a normally distributed error term. ANOVA tests and *t* tests were performed to decide the significance of each regression (*β* and *γ*) respectively the significance of each *X* effect (*β*). The normality assumption for each of the regressions was confirmed applying the Shapiro–Wilk W test for normality, the Shapiro-Francia W test for normality, as well as visual inspections of histograms for residuals (all tests are available from authors upon request) (38–40). Similarly, the linearity assumption for each of the regressions was confirmed applying visual inspections of scatter plots containing residuals against predicted values, respectively, *X*-values as specified in the comments to equations (1–4) (residual plots are available from authors upon request). The statistical software package Stata 18.0 SE Standard Edition was used for all the statistical descriptive and regression analysis (Stata Statistical Software: Release 18. College Station, TX: StataCorp LLC).

## Results

The summary statistics for the respondents in Table 1 show that the average age for the women was 61 years, the average height was 154 cm, the average weight was 78 kg, and the average BMI was 33 kg/m^2^ (even the 95% confidence interval of 32–34 was well into the obesity interval). The females’ fat percentage was 41% (32 kg), and the SMM percentage was 29% (22 kg) on average. HGS for the sampled women reached 19 kg. Average physical activity for the subjects of the study was just below 40 min daily, while sedentary behavior reached 9 h per day. The systolic blood pressure average was on the higher side with 123 mm Hg, while the diastolic was on the lower side with an average of 77 mm Hg. Average triglyceride level for the post-menopausal women was 145 mg/dL and HDL level was 59 mg/dL. Finally, WC of the female respondents was just above 100, both regarding the point estimate and the interval estimate.

**Table 1:** Participants’ body composition, physical activity and metabolic markers characteristics.

		Mean	SD	Lower CI	Upper CI
Education, %	1. Elementary	31	46	26	37
	2. High school	37	48	31	42
	3. University	29	46	24	35
	4. Postgraduate	3	16	1	5
Income, %	1. SR 1,000 – 2,999	13	33	9	17
	2. 3,000 - 5,999	11	32	7	15
	3. 6,000 - 9,999	21	41	16	26
	4. ≥ 10,000	55	50	49	61
Marital status, %	1. Married	70	46	64	75
	2. Divorced	10	31	7	14
	3. Widow	20	40	15	25
Social status, %	1. Live with husband only	4	19	1	6
	2. Live with children only	28	45	22	33
	3. Live with husband and children	63	48	57	68
	4. Live with others	1	9	0	2
	5. Live alone	5	22	3	8
Physical characteristics	Age (year)	61	7	60	62
	Height (cm)	154	5	153	154
	Weight (kg)	78	13	77	80
	BMI (kg/m2)	33	5	32	34
	Fat (kg)	32	8	31	33
	Fat percentage (%)	41	5	40	42
	SMM (kg)	22	3	22	23
	SMM percentage (%)	29	3	29	30
	FMR	1.44	0.31	1.40	1.48
	HGS (kg)	19	4	19	20
	HGS/BMI	0.59	0.15	0.58	0.61
	HGS/SMM	0.59	0.15	0.58	0.61
Physical activity	Physical activity (minutes/week)	274	229	246	301
	Sedentary (hours/day)	9	4	9	10
Metabolic bio markers	Triglyceride (mg/dL)	145	74	136	154
	HDL (mg/dL)	59	17	57	61
	Systolic (mm Hg)	123	16	122	125
	Diastolic (mm Hg)	77	8	76	78
	WC (cm)	103	11	102	104

BMI, body mass index; FMR, fat to muscle ratio; HGS, handgrip strength; SMM, skeletal muscle mass; HDL, high density lipoprotein; WC, waist circumference. mmHg, millimeter of mercury; cm, centimeter; mg/dL, milligrams per deciliter; kg/m^2^, kilogram per square meter.

The degree of linear association between measurements between age, body composition, physical activity, and metabolic markers was significant for different specific bivariate combinations (Table 2). Physical activity was positively associated with HGS (*r* = 0.16) and standardized HGS (HGS/BMI) (*r* = 0.14). Systolic blood pressure increases with age (*r* = 0.24). WC was the one most often associated with other measurements: age (*r* = 0.20); fat percentage (*r* = 0.66); SMM percentage (*r* = −0.63); FMR (*r* = 0.65); HGS/BMI (*r* = −0.49); HGS/SMM (*r* = −0.24). All other displayed correlations were statistically insignificant at the 5% significance level.

**Table 2:** Correlation coefficient between age and body composition, physical activity, and metabolic markers.

	Age (year)	Fat perc. (%)	SMM perc. (%)	FMR	HGS (kg)	HGS/BMI	HGS/SMM
Physical activity (minutes/week)	−0.0888	−0.0469	0.0473	−0.0294	0.1561*	0.1395*	0.1078
Sedentary (hours/day)	0.2241*	0.1082	−0.1315*	0.1420*	−0.1841*	−0.2739*	−0.1907*
Triglyceride (mg/dL)	0.0136	0.0456	0.0007	0.011	0.0204	−0.0465	−0.0674
HDL (mg/dL)	0.0423	−0.0869	0.057	−0.0597	−0.119	−0.0087	−0.0276
Systolic (mm Hg)	0.2358*	−0.0106	−0.0112	0.0193	−0.0776	−0.0992	−0.0683
Diastolic (mm Hg)	0.0442	−0.0305	0.0227	−0.0109	−0.0582	−0.0105	−0.0031
WC (cm)	0.1963*	0.6591*	−0.6255*	0.6543*	−0.0132	−0.4938*	−0.2373*

BMI, body mass index; FMR, fat to muscle ratio; HGS, handgrip strength; SMM, skeletal muscle mass; HDL, high density lipoprotein; WC, waist circumference. *p < 0.05.

The relationship between (standardized) HGS and age is displayed in Table 3: Higher age was significantly associated with lower HGS with a *p*-value of 0.007, while HGS/SMM did not exhibit a significant association with age (*p*-value = 0.83). We also report a visual overview about the relationship between (standardized) HGS and FMR in Table 3: Higher FMR did not exhibit a significant association with lower HGS, as shown by a p-value = 0.58. Lastly, we found a similar observation between FMR and HGS/SMM (*p*-value = 0.72) (Table 3).

**Table 3:** Relationship of age and FMR with HGS and HGS/SMM in the study population.

**Age**	**HGS**	
	R value	*p* value
	−0.1645	0.007
	**HGS/SMM**	
	R value	P value
	−0.013	0.831
**FMR**	**HGS**	
	R value	P value
	0.033	0.581
	**HGS/SMM**	
	R value	P value
	−0.022	0.717

HGS; handgrip strength, SMM; skeletal muscle mass, FMR; fat to muscle ratio.

In Table 4, OLS regressions of the types (1)–(4) mentioned above were carried out for HGS and HGS/SMM while controlling for age, respectively, FMR and then estimating the effects of different measurements related to physical activity, and biomarkers. Physical activity was positively associated with HGS, and the effect was nearly of the same magnitude (estimates 0.00243 and 0.00268) regardless of whether controlling for age or FMR. Higher sedentary levels negatively affect HGS (estimates −0.16 and − 0.19) and HGS/SMM (estimates −0.0092 and − 0.0089). Finally, WC did not affect HGS but negatively affects HGS/SMM in a statistically significant way, and with quite different magnitudes depending on whether one controls for age (*β* coefficient estimate of −0.00385) or controls for FMR (slope of −0.00614), e.g., higher WC is associated with reduced (standardized) HGS.

**Table 4:** Regression of HGS, respectively, HGS/SMM adjusted for age, respectively, FMR.

	HGS adjusted for		HGS/SMM adjusted for	
	Age	FMR	Age	FMR
Physical activity (minutes/week)	0.00243*	0.00268*	0.0000851	0.0000849
Sedentary (hours/day)	−0.156*	−0.194**	−0.00924**	−0.00894**
Triglyceride (mg/dL)	0.00118	0.00105	−0.000163	−0.000163
HDL (mg/dL)	−0.0250	−0.0262	−0.000281	−0.000300
Systolic (mm Hg)	−0.0103	−0.0196	−0.000804	−0.000791
Diastolic (mm Hg)	−0.0241	−0.0273	−0.0000547	−0.0000725
WC (cm)	0.00674	−0.0210	−0.00385***	−0.00614***

FMR, fat to muscle ratio; HGS, handgrip strength; SMM, skeletal muscle mass; HDL, high density lipoprotein; WC, waist circumference.*p < 0.05 **p < 0.01; ***p < 0.001.

## Discussion

Our study showed that physical activity and sedentary behavior were associated with HGS, HGS/BMI and/or HGS/SMM. Considering age and FMR in the regression, physical activity and sedentary behavior were associated with HGS, while sedentary behavior and WC were associated with HGS/SMM. The adjustments to body composition compartments help to assess the contribution of fat and muscle mass in these relationships. Longitudinal and follow-up studies are warranted to examine the impact of lifestyle changes on muscle function and the estimation of sarcopenia.

The average HGS was 19.2 ± 3.9 kg for the current sample of post-menopausal women, which was consistent with other studies that have similar gender and age groups (Charlotte (41–45)). However, it was slightly lower than the values of HGS for older adults in Saudi Arabia. The mean values of HGS for women in this study were 23.2 ± 4.7 kg for the younger old group (65–69 years), 21.1 ± 4.6 kg for the middle group (70–74 years), and 18.8 ± 4.9 kg for the oldest group (75–80 years) (46). Some studies reported lower values of HGS compared to our study (47–50). We stress the importance of HGS as it has a fundamental relationship with the possibility of reducing disability (51–56). Women had significantly lower grip strength than men, which indicates the importance of increasing awareness among women about sports activities, especially activities that increase muscle mass and strength (57).

There were relationships between increased physical activity and decreased sedentary behavior with increased HGS. The relationship with physical activity seems to be partially attributed to the increase in skeletal muscle mass. In line with this, several studies supported the hypothesis that higher levels of physical activity were associated with decreased sarcopenia (58–60). Similarly, a direct inverse association between the level of physical activity and HGS is reported in old age (61). Similarly, low-grade, steady-state exercise improves HGS and the functioning of limb muscles in older adults with sarcopenia (62). The exercise therapies in these participants included non-specific, home-based regular exercises and physical activities (62), which are party identical to the physical activities regimens used in our study. Sánchez-Sánchez et al. (63) indicated that when sedentary behavior or even light physical activity was replaced by MVPA, this change reduced the incidence of sarcopenia. Bell et al. (64) reported that too few steps during the day and inactive behavior lead to impaired muscle protein synthesis and reduced muscle mass as well as strength. Two groups were compared regarding their degree of sedentary behavior (less than 4 h/day) and (at least 11 h/day). The authors noted the probability of developing sarcopenia increased 2.14 times in the sedentary group, and indicated that an increase of one hour of sedentary behavior each day was associated with a risk 1.06 times higher of developing sarcopenia (65). Taken together, these results indicate that higher levels of sedentary behavior were associated with higher levels of sarcopenia.

Muscle weakness is partly a consequence of muscle atrophy due to a reduction in the amount of muscle contractile machinery. However, contractile dysfunction may also contribute to muscle weakness independent of muscle mass (66). Therefore, the reporting of raw HGS may not adequately represent the intrinsic quality of skeletal muscle. The HGS/SMM adjusts for muscle mass, which may be a more accurate representation of the intrinsic force-generating properties of skeletal muscle. We found negative correlations of HGS/SMM with the sedentary activities of the study participants. This observation is consistent with the reports of muscle weakness in sedentary participants, which partly involves contractile dysfunction independent of muscle mass (67). We also observed a negative correlation of WC with HGS/SMM, which confirms and extends the previous observations of the adverse effects of body fats on muscle contractile apparatus (68). The adjustment for age and FMR did not affect the inverse correlations of HGS/SMM with a sedentary lifestyle and WC. Together, these observations suggest that a sedentary lifestyle and obesity can negatively affect muscle contractile machinery independent of muscle mass, age, and FMR.

Obesity was high among the participants (fat percentage > 40% body weight), leading to increased WC (> 102 cm), which was coupled with low HGS/SMM. This means fat mass directly affects muscle strength independent of the mass of skeletal muscle. The abdominal fats associated with WC are considered bad fats and can cause reduced HGS/SMM through several potential mechanisms. Increased body fats cause release of inflammatory cytokines, which negatively affect skeletal muscle mass and strength (69). A higher WC and obesity also disrupt muscle oxidative balance by increasing oxidative stress (70), which is a well-established trigger of muscle weakness and low HGS (71). Increased amount of body fats also increases the levels of stress hormones (69), which blunt anabolism and activate catabolism of skeletal muscle, leading to muscle weakness and atrophy. Lastly, obesity also inhibits muscle satellite cells (72), causing reduced muscle repair and regeneration following myotoxic injuries.

We used FMR as index for fat mass, which may also be relevant for investigating sarcopenia and sarcopenic obesity. A study by Stefanaki and colleagues (73) revealed that increased fat deposition was negatively correlated with muscle and bone density, reflective of a phenomenon known as osteosarcopenic obesity. As an aging disease, sarcopenia is known to be associated with various metabolic disorders, including obesity (74). Due to the correlation between sarcopenic obesity and functional impairment, morbidity, and mortality, addressing sarcopenic obesity among the aging population is a priority (75, 76). Previous studies have shown that the effects of aging are greater when sarcopenia is associated with metabolic syndrome (77, 78). It should be noted that not all previous studies found an association between abdominal fat and sarcopenia. For example, some studies reported that sarcopenia is associated with high WC (79–81), but other studies found a negative relationship between WC and sarcopenia (82, 83), and others found no relationship between WC and sarcopenia (84).

Interestingly, we did not find a significant association of absolute HGS with FMR in post-menopausal women. Similar findings were observed after adjusting HGS to SMM. These observations suggest that the FMS may not be a primary dictator of absolute or adjusted HGS. While this observation appear counterintuitive, previous reports of a dissociation between body fat mass absolute HGS in overweight women align with our findings (85). Conversely, body fat mass may be a prime determinant of handgrip endurance (85). However, the FMR in our study cohort was limited to an FMR of ≈2.3. We cannot rule out a possible inverse correlation of FMR with HGS in extremely obese people. This is partly because of the well-established negative effects of body fat mass on skeletal muscle strength (86).

We used an HGS threshold of 16 kg for diagnosis of probable sarcopenia. This threshold is defined by the European Working Group on Sarcopenia in Old People and The Foundation of the National Institute of Health (87). On the other hand, the Asian Working Group for Sarcopenia uses an HGS threshold of 18 kg for defining sarcopenia (87). Therefore, it is possible that using a different HGS threshold for defining sarcopenia may partly change our findings. Another limitation of the study is that we did not investigate hormone therapy intake, and future studies are advised to examine it because prolonged use of hormone therapy among post-menopausal women resulted in high muscle mass and low prevalence of sarcopenia (88). Moreover, the current study is a cross-sectional design, using a convenient sampling at a single center that has a gym and walking track, such that the current sample may be those women who want to improve their fitness and lose weight. It was not clear when participants had started their physical activity program to confirm its impact on muscle mass and strength. Lastly, information regarding lifestyle and muscle strength in post-menopausal women is very limited, and this paper will be a reference value for future research. Longitudinal studies are warranted and highly needed to examine the phenomenon of sarcopenia and the role of lifestyle among the post-menopausal women in Saudi Arabia.

## Conclusion

Considering age, FMR, and SMM, we report that high physical activity, low sedentary behavior, and low WC are negatively associated with a risk of low HGS among Saudi post-menopausal women.

## Data Availability

The raw data supporting the conclusions of this article will be made available by the authors, without undue reservation.
